# Anxiety in patients with hyperthyroidism

**DOI:** 10.1192/j.eurpsy.2022.491

**Published:** 2022-09-01

**Authors:** S. Nevzorova

**Affiliations:** Kharkiv National Medical University, Department Of Psychiatry, Narcology, Medical Psychology And Social Work, Kharkiv, Ukraine

**Keywords:** Anxiety, Mental symptoms, Thyrotoxicosis, Hyperthyroidism

## Abstract

**Introduction:**

Mental symptoms are the first manifestations of hyperthyroidism. They include anxiety, dysphoria, irritability, emotional lability, sleep disorders, intellectual dysfunction, mania or depression. Anxiety is the main symptom and requires more detailed study.

**Objectives:**

The objective was to determine symptomatology of anxiety in patients with hyperthyroidism and compare with euthyroid patients.

**Methods:**

The study included 56 patients with hyperthyroidism (high free T3 and free T4, suppressed TSH) and 32 euthyroid patients (normal free T3, free T4 and TSH) of the control group. For psychiatric assessment State-Trait Anxiety Inventory [STAI], Hamilton Depression Rating Scale [HAM-D], and Hamilton Anxiety Rating Scale [HAM-A] were used.

**Results:**

Total scores obtained from STAI, HAM-D and HAM-A were significantly greater in the hyperthyroidism group than that of the euthyroid group (p<0.05). The level of state anxiety in patients with hyperthyroidism was 51.39 ± 0.95 (high level) compared with 41.59 ± 2.41 (moderate level) in the control group. The level of trait anxiety in patients with hyperthyroidism was 46.86 ± 0.69 (high level), and 44.16 ± 2.17 (moderate level) in the control group. Psychomotor agitation (HAM-D # 9), psychic anxiety (HAM-D # 10), insomnia (HAM-A # 4) and weight loss (HAM-D # 16) were typical for patients with hyperthyroidism, while in the control group predominate the feelings of fatigue, weakness and loss of interest in working (HAM-D # 7).

**Conclusions:**

The prevalence of anxiety in patients with hyperthyroidism is significantly more frequent compared to euthyroid patients. Anxiety and other psychiatric symptoms should be considered by both endocrinologists and psychiatrists.

**Disclosure:**

No significant relationships.

